# Cannabigerol Activates Cytoskeletal Remodeling via Wnt/PCP in NSC-34: An In Vitro Transcriptional Study

**DOI:** 10.3390/plants12010193

**Published:** 2023-01-03

**Authors:** Ivan Anchesi, Federica Betto, Luigi Chiricosta, Agnese Gugliandolo, Federica Pollastro, Stefano Salamone, Emanuela Mazzon

**Affiliations:** 1 IRCCS Centro Neurolesi “Bonino-Pulejo”, Via Provinciale Palermo, Contrada Casazza, 98124 Messina, Italy; 2 Department of Pharmaceutical Sciences, University of Eastern Piedmont, Largo Donegani 2, 28100 Novara, Italy

**Keywords:** cannabigerol, NSC-34, axon guidance, WNT/planar cell polarity (PCP) signaling, Ephrin-Eph signaling

## Abstract

Cannabigerol (CBG) is a non-psychoactive phytocannabinoid present in the *Cannabis sativa* L. plant. In our study, CBG at the concentration of 10 µM was used to treat NSC-34 motor neuron-like cells. The aim of the study was to evaluate the effects of CBG on NSC-34 cells, using next-generation sequencing (NGS) technology. Analysis showed the activation of the WNT/planar cell polarity (PCP) pathway and Ephrin-Eph signaling. The results revealed that CBG increases the expression of genes associated with the onset process of cytoskeletal remodeling and axon guidance.

## 1. Introduction

**Figure 1:**
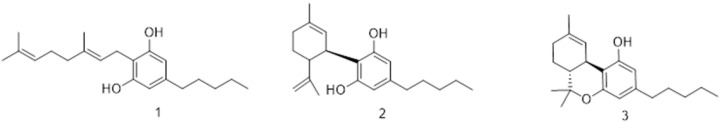
Structure of Cannabigerol (1), Cannabidiol (2), and Δ^9^-tetrahydrocannabinol (3).

*Cannabis sativa* is an ancient plant, whose oldest and most famous use is in medicine [1]. This plant represents a reservoir of over 100 cannabinoids, and cannabigerol (CBG) is one of these. CBG, a non-psychoactive phytocannabinoid, is the main actor involved in most of the medical effects of cannabis, as well as being the precursor to Δ^9^-tetrahydrocannabinol (Δ^9^-THC), cannabidiol (CBD), and many others (Figure 1). CBG has been shown to have a therapeutic effect on many neurodegenerative diseases [2,3,4]. The molecular targets of CBG are poorly characterized. CBG is an agonist of alpha (2)-adrenoceptors and moderately block 5-hydroxytryptamine (5HT_1A_) receptors [5], and it interacts with different receptors, such as transient receptor potential cation channels (TRPV) [6,7] and peroxisome proliferator-activated receptor gamma (PPARγ) [6]. Moreover, CBG shows a binding affinity for cannabinoid receptor 1 (CB1) and cannabinoid receptor 2 (CB2). CB receptors are G protein-coupled receptors (GPCRs) expressed in important brain regions and are associated with several neurological processes. CBG seems to be a partial agonist of both CBs; moreover, binding assays have demonstrated that CBG could also act as an allosteric modulator for CB1 [8]. Several pieces of evidence have demonstrated the role of natural compounds extracted from *C. sativa* in neurogenesis [9,10] and neuroprotection [11,12], and our team have previously highlighted the ability of CBG to influence synaptic pathways [13,14]. Neurons regulate their cytoskeleton to achieve changes in shape, migration, neurites extension, and polarization [15]. Furthermore, axon guidance is an essential process for the nervous system in order to ensure the integrity and accuracy of the nerve patterning [16]. 

NSC-34 cells are hybridoma cells of neuroblastoma and motor neuron-enriched spinal cord cells. A previous study demonstrated that CBG treatment was efficient in promoting survival signaling, as well as inducing regeneration in differentiated motor neuron-like cells [17]. Considering this, we exposed undifferentiated NSC-34 cells to CBG for 24 h at a concentration of 10 µM. Transcriptomic analysis was performed to evaluate the activation of signaling pathways related to cytoskeletal remodeling and axon guidance after CBG treatment.

## 2. Results

### 2.1. MTT Assay

**Figure 2:**
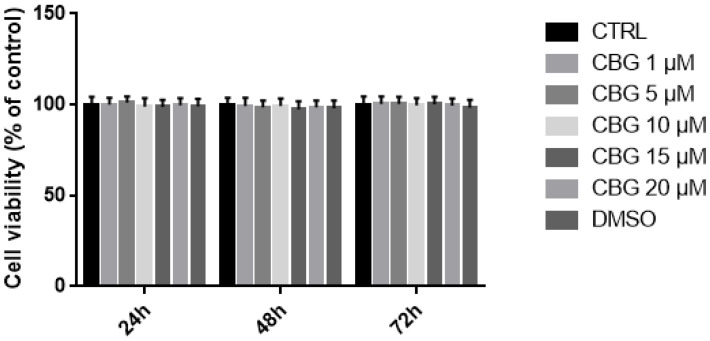
Evaluation of possible effects of CBG at concentrations of 1, 5, 10, 15, and 20 µM, and DMSO on NSC-34 cells viability at 24 h, 48 h, and 72 h. No significant difference in cell viability was detected.

MTT assay was performed to evaluate the effects of CBG in the concentration range of 1–20 µM on cell viability. The results showed that CBG at the tested concentrations at different time points—namely, 24 h, 48 h, and 72 h—did not decrease NSC-34 cell viability. We also analyzed DMSO toxicity, given that CBG was dissolved in DMSO. However, DMSO was not toxic at the concentrations used (Figure 2).

### 2.2. Transcriptomic Analysis

**Figure 3:**
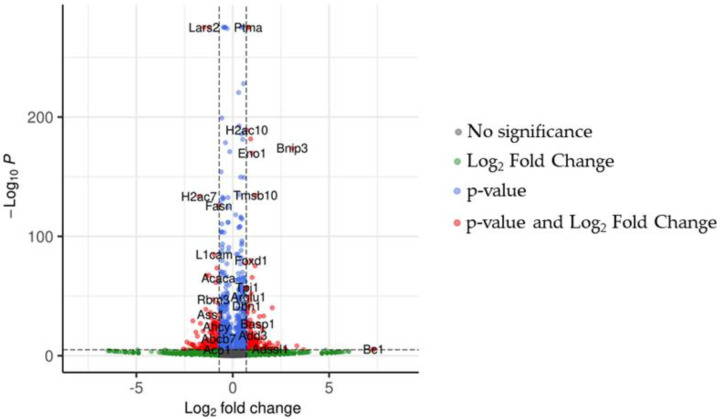
The volcano plot shows the behavior of genes in our analysis. Red and blue dots are the genes with *p*-values lower than 0.05. Additionally, genes represented by red dots have a fold change higher than 0.7 orders of magnitude. The green dots highlight genes with fold changes over the threshold but, along with the grey dots, without *p*-value significance.

In treated NSC-34 cells, we focused our analysis on differentially expressed genes (DEGs) between CBG and CTRL groups. The volcano plot in Figure 3 shows the distribution of DEGs in the analysis.

After q-value correction, the analysis revealed 4937 DEGs compared to CTRL. The expression levels of markers associated with CB1 signaling were evaluated to assess the status of the cells after treatment (Table 1). CBG has an affinity to CB1, and indeed, up-regulation of the *Cnr1* gene and of the DEGs of CB1 signals did occur (Table 1). CBG exhibits affinity with several receptors, but our analysis did not show any variation of genetic expression between them. 

Subsequently, the study analyzed the WNT/planar cell polarity (PCP) signaling. As seen in Table 2, cells treated with CBG experienced up-regulation of many genes of this pathway. These data suggest CBG involvement in WNT/PCP-dependent cytoskeleton remodeling.

Analysis of DEGs associated with the Ephrin-Eph signaling showed up-regulation of different genes relative to the Ephrin pathway, suggesting that this signaling was affected by the CBG treatment (Table 3).

## 3. Discussion

NSC-34 is a hybrid cell line produced via the fusion of motor neuron-enriched embryonic mouse spinal cord cells with mouse neuroblastoma. We assessed, through MTT assay (Figure 2), that CBG at a concentration of 10 µM did not affect NSC-34 cells’ viability.

In the biosynthesis of cannabinoids, both CBD and Δ^9^-THC derive from CBG, with the latter having the lower degree of unsaturation on the terpenyl moiety. CBD and Δ^9^-THC have the same molecular formula and almost identical molecular weights of 314.464 g/mol and 314.469 g/mol, respectively. The most important structural difference between the two major cannabinoids is represented by the electrophilic cyclization step in the biosynthetic pathway, in which CBD is generated from the loss of a proton to obtain the exocyclic double bond, leaving the hydroxyl group free. Instead, Δ^9^-THC is characterized by a pyran ring due to the closure of the phenolic hydroxyl with the menthyl moiety (Figure 1). This small divergence in their structure is responsible for the entirely different pharmacological properties [18].

Transcriptomic analysis showed up-regulation of the gene *Cnr1*, which encodes Cannabinoid receptor 1 (CB1). Our results showed differentially expressed genes involved in Wnt “non-canonical” pathways. Differing from canonical pathways that lead to β-catenin dependent transcription, non-canonical Wnt is independent of β-catenin. The influences of Δ^9^-THC and CBD on the Wnt/β-catenin canonical pathway has already been documented. CBD has been shown to mediate a neuroprotective effect through the Wnt/β-catenin canonical pathway [19]. The effect of Δ^9^-THC on this pathway has already been documented with a combination of active compounds [20], but there is no evidence regarding its effect as a pure compound. In addition, the effect of Δ^9^-THC and CBD on Wnt-non canonical signaling has not been documented. Our analysis revealed up-regulation of different genes belonging to the non-canonical Wnt/PCP pathway, which refers to the organization of cells within the plane of a cell sheet [21]. 

**Figure 4:**
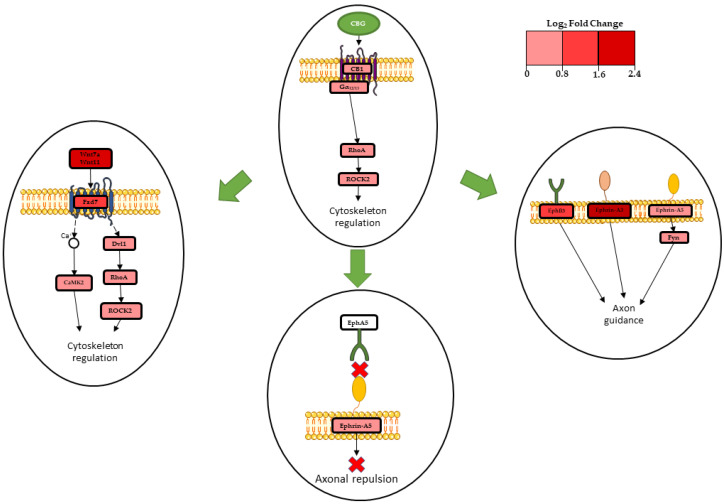
Mechanisms involved in the cytoskeletal remodeling of NSC-34 after CBG treatment. The figure represents pathways involved in the process. A signal influenced by CBG is represented in each oval. Green arrows indicate influence of CB1 activation. Continuous arrows indicate direct associations, while dashed arrows indicate indirect association. A red X indicates that there is no interaction between the receptor and ligand. A red palette indicates genes log_2_ Fold Change. The results of transcriptomic analysis suggest that CBG induces initial cytoskeletal remodeling of NSC-34 by WNT/planar cell polarity (PCP) signaling, Ephrin-Eph signaling, and the involvement of CB1.

In fact, Wnt11 (*Wnt11*) and Wnt7a (*Wnt7a*), which are non-canonical Wnt isoforms, were strongly upregulated after treatment with CBG (Table 2). Previous studies have demonstrated that Wnt11 suppresses both the expression of β-catenin and represses the effect of the canonical Wnt3a [22,23]. Wnt7a is expressed in the central nervous system, where it stimulates synapse formation and regulates dendritic spinal growth [24]. Frizzled receptors (*Fzd*), in particular *Fzd3*, *Fzd6*, and *Fzd7*, exhibited up-regulation (Table 2), and these are GPCRs involved in canonical and non-canonical Wnt pathway activation [21]. Our transcriptome demonstrated up-regulation of *Fzd3* and *Fzd6*, which have already been found to be involved in non-canonical Wnt signaling [25]. *Fzd7* is particularly relevant to neurons; in fact, it is documented as the post-synaptic membrane receptor of Wnt7a [26], and it is able to directly promote the growth of dendritic spines [21]. Wnt7a- and Wnt11-binding Frizzled-7 activates the downstream cascade of the WNT/planar cell polarity (PCP) signaling, which strongly influences the mechanism of cytoskeleton regulation, as described in Figure 4.

*Celsr1*, *Celsr2*, and *Celsr3* codifying for the Cadherin EGF LAG seven-pass G-type receptor 1, *Ankrd6*, *Dvl1*, and *Prickle3* exhibited up-regulation (Table 2). The proteins codified by these genes are involved in the Wnt/PCP pathway. These proteins interact by assembling two different complexes: one formed by Fzd, Dvl, and ANKRD6; and the second one formed by VANGL and Prickle. The asymmetrical distribution of these complexes at opposite poles of the cell generates polarity [21]. *Prickle3* was strongly upregulated. This gene codifies for a protein that localizes at the mitotic spindle poles and centrioles, and the gene has a role in PCP process [27]. Both of the PCP complexes interact with Celsr protein, which regulates the establishment of PCP and is involved in axon guidance and neurites morphogenesis [28]. Dvl1 is an important scaffold protein, fundamental for the transmission of the signal of the non-canonical Wnt pathway [21]. It is also expressed at the level of dendritic growth cones, and it is crucial in dendrite formation and maturation [29]. Ferrari et al. demonstrated that *Dvl1* exerts its role when *Wnt7a* is expressed; when both *Dvl1* and *Wnt7a* are expressed they have a significant impact on regulating dendritic morphogenesis [30]. *CaMK2b*, *CaMK2d*, *CaMK2a*, and *CaMK2g*, each codifying for different isoforms of calcium/calmodulin-dependent protein kinase type II (CaMKII), were up-regulated (Table 2). These protein kinases play a structural role in the reorganization of the actin cytoskeleton during plasticity by grouping actin filaments [31].Their activation strongly depends on the presence of *Fzd7*, and even more so on *Dvl1* expression, promoting dendritic tree development [30] and regulating actin cytoskeleton [32]. 

In addition, our analysis showed further effectors on the Wnt/PCP pathway; namely, *RhoA* and *Rock2* genes showed a high level of expression (Table 2). RhoA is a GTPase that acts upon the Rho associated kinase 2, also called ROCK2 [33].

The gene *Gna13* exhibited up-regulation (Table 1). This gene codifies for the CB1-coupled Gα_13_ subunit. Gα_13_ participates in RhoA/ROCK2 signaling [34,35], which is important for actin cytoskeleton regulation [36]. Cell shape modifications are also involved in axonal morphogenesis and axon guidance [37], and previous evidence has demonstrated that the activation of CB1 can promote axon elongation and outgrowth [38].

Our analysis showed up-regulation of the gene *Efna5*, codifying for ephrin-A5 ligand; strong up-regulation of *Efna3*, codifying for ephrin-A3 ligand; and strong up-regulation of *Ephb3*, codifying for the ephrin receptor EphB3 (Table 3). These genes are involved in Ephrin-Eph signaling, which is involved in neuronal development, axon guidance, and axonal growth [39]. In our analysis, *Eph5*, codifying for EphA5 receptor, was not differentially expressed. Castellani et al. demonstrated that when the ligand ephrin-A5 binds to a EphA5 receptor, ephrin-A5 acts as a repulsive axonal guidance signal [40]. Therefore, our results indicate that in NSC-34 treated with CBG, there is not an axonal repulsion signal due to ephrin-A5 (Figure 4). In addition, our analysis showed up-regulation of protein β1-integrin (*Itgb1*) (Table 3). This protein interacts with ephrin-A5, mediating cellular adhesion and neurite growth [41]. The Src-family kinase Fyn (*Fyn*), which is involved in ephrin-A5 signaling inducing axon guidance [42], demonstrated up-regulation (Table 3). *Efna3*, codifying for the ephrin-A3 ligand, was up-regulated and has been shown to act as an axonal repulsive signal [43]. Lastly, our analysis showed up-regulation of *Ephb3*, codifying for the EphB3 receptor. This receptor is able to promote axon guidance in vitro [44,45]. Transcriptomic data showed a connection between the Wnt/PCP pathway and Ephrin-Eph signaling. Additionally, the protein Celsr3, previously mentioned as being involved in non-canonical signaling, interacts with ephrin-A5 in regulating axon guidance [46]. Transcriptomic analysis showed that treatment of NSC-34 cells with CBG (10µM) induces cytoskeletal remodeling and axon guidance. These processes are promoted via CB1 receptor involvement, activation of Wnt/PCP pathway, and Ephrin-Eph signaling, as summarized in Figure 4. These results are promising, opening the door to further study that could be useful in proving the efficacy of cannabigerol. 

## 4. Materials and Methods

### 4.1. CBG Extraction from Cannabis Sativa and Isolation

CREA-CIN (Rovigo, Italy) was the provider of *Cannabis sativa* var. Carma, obtained from greenhouse cultivation, where a voucher specimen is kept. The manipulation of the plant was performed according to the authorization SP/106 23/05/2013 of the Ministry of Health (Rome, Italy). CBG extraction was performed by heating 1 Kg of dried, powdered, flowered aerial parts at 120 °C for 2.5 h, in order to obtain precannabinoid decarbossilation, and was then extracted using acetone (2 × 9 L). Subsequently, the solvent was removed and the remaining black resinous residue (74 g, 7.4%) was dissolved in methanol (30 mL/g of extract). Waxes and pigments were filtered using through RP C-18 silica gel. Once the methanol had evaporated, 36 g of a dark green extract was obtained through the evaporation of methanol and was further purified through gravity column chromatography on silica gel 60 (70–230 mm). From purification, 75 g of petroleum ether-EtOAc, 8:2, as eluent, to afford 5 g of a yellow oil, which was then crystallized with petroleum ether to obtain 3 g of pure CBG (0.3%). The JEOL ECP 300—300 MHz spectrometer (JEOL, Pleasanton, CA, USA) was used to measure ^1^H-NMR spectra. The ^1^H (400 MHz) and ^13^C (100 MHz) NMR spectra were measured on Bruker 400 spectrometers (Bruker^®^, Billerica, MA, USA). Chemical shifts were measured in reference to the residual solvent signal (CDCl_3_: δ_H_: 7.26, δ_C_: 77.16). (Supplementary Materials, Figures S1 and S2). 

Waxes and pigments were eliminated using Reverse phase (RP) C-18 (POLYGOPREP60-30 C18). TLC on Merck 60 F254 (0.25 mm) plates, which were visualized by UV inspection and/or by spraying with 5% H_2_SO_4_ in ethanol and heating, were used to monitor CBG purification.

### 4.2. Cell Culture and Treatment

NSC-34 cell lines were provided by the Cedarlane Corporation (Burlington, ON, Canada) and were maintained in DMEM High Glucose, consisting of 10% Fetal Bovine Serum, 1% penicillin/streptomycin, and 1% L-Glutamine. All reagents were provided by Sigma-Aldrich, Merck KGaA (Darmstadt, Germany). The cells were kept in a cell incubator at 37° C with 5% CO_2_. Cells were seeded in 6-well plates and 96-well plates at a density of 125.000 cells/mL. The CBG treatment was performed at a concentration of 10 µM (3.16 µg/mL) in DMSO (<0.1%) for 24 h. Control wells equally underwent a complete medium change. At the end of the treatment, cells in 6-well plates were harvested to perform the transcriptomic analysis, while those in 96-well plates underwent MTT assay. 

### 4.3. Proliferation Assessment Using MTT

To evaluate the effects on cell viability of CBG, NSC-34 cells were cultured in 96-well plates and incubated with different CBG doses (1, 5, 10, 15, and 20 µM) for different durations (24 h, 48 h, and 72 h). Given that the CBG was dissolved in DMSO, we also included cells treated with DMSO at the major concentration used (DMSO < 0.1%). At the end of the treatment, cells underwent a full medium change, with the medium having been previously prepared by adding MTT at concentration of 0.5 mg/mL (Sigma-Aldrich Merck KGaA (Darmstadt, Germany). After 4 h in an incubator at 37 °C, the crystals obtained by the reaction were resuspended in acid isopropanol. After a brief mixing, the optic density was measured using a spectrophotometer at 570 nm.

### 4.4. Statistical Data Analysis

Statistical analysis was carried out using GraphPad Prism version 6.0 software (GraphPad Software, La Jolla, CA, USA). The data were statistically analyzed via two-way ANOVA tests and Bonferroni post hoc tests for multiple comparisons. A *p*-value of less than or equal to 0.05 was considered statistically significant. Results are reported as mean ± SD of N experiments.

### 4.5. Total RNA Extraction and Library Preparation

After 24 h of treatment with CBG at a concentration of 10µM, the cells were harvested and centrifugated, and the supernatant was discarded. The pellet was used for RNA extraction, along with the Maxwell^®^ RSC simplyRNA Cells Kit (Promega, Madison, WI, USA), which was used followed the manufacturer’s instructions. TruSeq RNA Exome protocol (Illumina, San Diego, CA, USA) was used to perform library preparation, in compliance with the manufacturer’s instructions. 

### 4.6. Transcriptomic Analysis and Bioinformatics Inspection

The obtained libraries were then sequenced with an Illumina MiSeq Instrument. We checked the quality of the raw data of the samples of CTRL and CBG at 10 µM using FastQC 0.11.4 (Babraham Institute, Cambridge, UK). Adapters and the poor-quality bases were removed using Trimmomatic 0.40 (Usadel Lab, Aachen, Germany) [47]. The GRCm39 version of the mouse reference genome was chosen, along with the primary transcript assembly M28, to perform the alignment with Spliced Transcripts Alignment to a Reference (STAR) RNA-seq aligner 2.7.10a (New York, NY, USA) [48] and transcript counting with the python package htseq-count 0.13.5 (European Molecular Biology Laboratory (EMBL), Heidelberg, Germany) [49]. Using R 4.2.0 (R Core Team) and the DESeq2 library 1.36.0 [50], we analyzed the DEGs of the CTRL against the 10 µM groups. We did not apply any fold-change cutoff, but we removed the false-positive DEGs (q-value higher than 0.05) using the post-hoc Benjamini–Hochberg procedure. In order to proceed with the identification of the biological role of CBG, we used the Kyoto Encyclopedia of Genes and Genomes (KEGG) database [51]. In detail, we paid attention to the specific cluster of genes observed in the pathways “Axon Guidance” (mmu04360) and “Wnt signaling pathway” (mmu04310).

## 5. Conclusions

Our analysis confirms that CBG increases the expression of genes involved in the WNT/planar cell polarity (PCP) pathway, such as *Wnt11*, *Wnt7a*, *Fzd7*, *Dvl1*, *Rock2*, and the genes codifying for CAMKII isoforms. In addition, CBG increases the expression of genes associated with ephrin-Eph, such as *Efna3*, *Efna5*, and *Ephb3*. Transcriptomic analysis demonstrated that CBG treatment of NSC-34 promotes cytoskeletal remodeling, stimulates dendrites outgrowth, and promotes axon guidance.

## Figures and Tables

**Table 1:** Differentially expressed genes involved in CB1 signaling.

Genes	CTRL vs. CBG Treated Fold Change	CTRL vs. CBG Treated log_2_ Fold Change	CTRL vs. CBG Treated *p*-Value	CTRL vs. CBG Treated q-Value
*Cnr1*	1.60	0.68	2.26 × 10^−16^	4.74 × 10^−15^
*Gna13*	1.10	0.15	5.43 × 10^−4^	2.17 × 10^−3^

The fold change for the analysis of CTRL vs. treated cells was computed as CBG treated/CTRL. Both the arithmetic fold change and normalized log_2_ value are provided in the table. All values are rounded to the second decimal.

**Table 2:** Differentially expressed genes involved in WNT/planar cell polarity (PCP) signaling.

Genes	CTRL vs. CBG Treated Fold Change	CTRL vs. CBG Treated log_2_ Fold Change	CTRL vs. CBG Treated *p*-Value	CTRL vs. CBG Treated q-Value
*Fzd3*	1.18	0.24	1.33 × 10^−4^	6.24 × 10^−4^
*Fzd6*	1.67	0.74	1.69 × 10^−2^	4.08 × 10^−2^
*Fzd7*	2.12	1.09	7.26 × 10^−3^	2.00 × 10^−2^
*Celsr1*	2.37	1.25	2.02 × 10^−2^	4.73 × 10^−2^
*Celsr2*	1.21	0.28	2.99 × 10^−3^	9.35 × 10^−3^
*Celsr3*	1.1	0.15	1.06 × 10^−2^	2.74 × 10^−2^
*Dvl1*	1.35	0.44	3.16 × 10^−11^	4.16 × 10^−10^
*Prickle3*	5.46	2.45	1.64 × 10^−3^	5.59 × 10^−3^
*Ankrd6*	2.23	1.16	4.11 × 10^−3^	1.23 × 10^−2^
*Rhoa*	1.47	0.56	2.01 × 10^−68^	2.65 × 10^−66^
*Wnt7a*	5.02	2.33	2.99 × 10^−3^	9.35 × 10^−3^
*Wnt11*	4.56	2.19	6.85 × 10^−4^	2.66 × 10^−3^
*Camk2a*	1.43	0.52	2.99 × 10^−5^	1.63 × 10^−4^
*Camk2b*	1.55	0.64	1.16 × 10^−4^	5.52 × 10^−4^
*Camk2d*	1.18	0.24	1.97 × 10^−8^	1.86 × 10^−7^
*Camk2g*	1.13	0.18	4.33 × 10^−3^	1.29 × 10^−2^
*Rock2*	1.16	0.22	5.67 × 10^−9^	5.67 × 10^−8^

The fold change for the analysis of CTRL vs. treated cells was computed as treated/CTRL. Both the arithmetic fold change and normalized log_2_ value are provided in the table. All values are rounded to the second decimal.

**Table 3:** Differentially expressed genes involved in Ephrin-Eph signaling.

Genes	CTRL vs. CBG Treated Fold Change	CTRL vs. CBG Treated log_2_ Fold Change	CTRL vs. CBG Treated *p*-Value	CTRL vs. CBG Treated q-Value
*Efna3*	3.13	1.65	1.43 × 10^−9^	1.56 × 10^−8^
*Efna5*	1.10	0.14	1.08 × 10^−2^	2.80 × 10^−2^
*Ephb3*	2.34	1.23	6.73 × 10^−3^	1.88 × 10^−2^
*Itgb1*	1.15	0.21	1.07 × 10^−11^	1.48 × 10^−10^
*Fyn*	1.22	0.29	2.46 × 10^−9^	2.58 × 10^−8^

The fold change for the analysis of CTRL vs. treated cells was computed as treated/CTRL. Both the arithmetic fold change and normalized log_2_ value are provided in the table. All values are rounded to the second decimal.

## Data Availability

The data presented in this study are openly available in the NCBI Sequence Read Archive at BioProject accession numbers PRJNA860777, PRJNA839187.

## Supplementary Materials

The following supporting information can be downloaded at: https://www.mdpi.com/article/10.3390/plants12010193/s1, Figure S1: ^13^C NMR of CBG in CDCl_3_ 400 MHz, Figure S2: ^1^H NMR of CBG in CDCl_3_ 400 MHz.
