# Supplementary figures and images for: Cannabigerol Activates Cytoskeletal Remodeling via Wnt/PCP in NSC-34: An In Vitro Transcriptional Study
Source: Plants (Basel). 2023 Jan 3;12(1):193. doi: 10.3390/plants12010193 (PMC9823669; doi:10.3390/plants12010193)

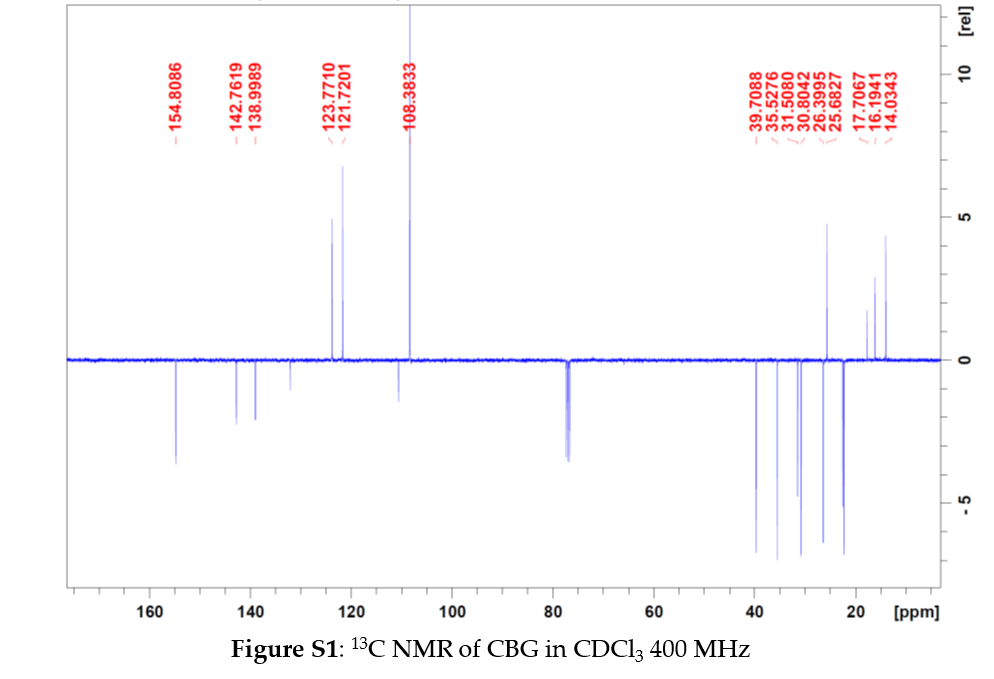

Supplement: Supplementary file 1 [file plants-12-00193-s001.zip › Figure S1.png]

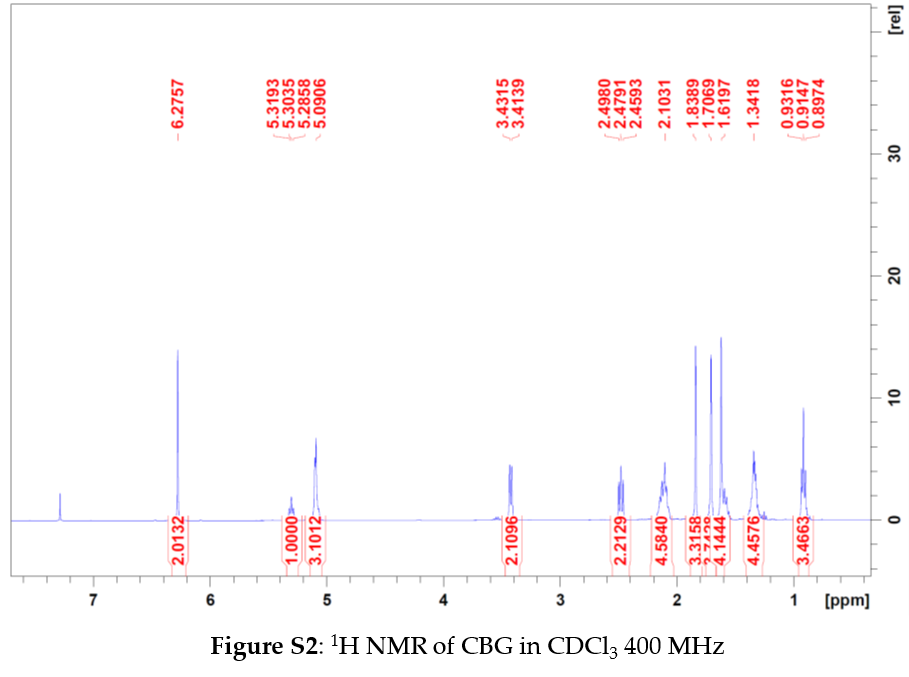

Supplement: Supplementary file 1 [file plants-12-00193-s001.zip › Figure S2.png]
